# The impact of COVID-19 on physical activity behaviour in Italian primary school children: a comparison before and during pandemic considering gender differences

**DOI:** 10.1186/s12889-021-12483-0

**Published:** 2022-01-08

**Authors:** Laura Dallolio, Sofia Marini, Alice Masini, Stefania Toselli, Rita Stagni, Maria Cristina Bisi, Davide Gori, Alessia Tessari, Alessandra Sansavini, Marcello Lanari, Laura Bragonzoni, Andrea Ceciliani

**Affiliations:** 1 Department of Biomedical and Neuromotor Sciences, University of Bologna, 40126 Bologna, Italy; 2 Department of Electrical, Electronic, and Information Engineering “Guglielmo Marconi” University of Bologna, 40136 Bologna, Italy; 3 Department of Psychology “Renzo Canestrari”, University of Bologna, 40127 Bologna, Italy; 4 Pediatric Emergency Unit, S. Orsola University Hospital, Scientific Institute for Research and Healthcare (IRCCS) Azienda Ospedaliero-Universitaria di Bologna, 40138 Bologna, Italy; 5 Department for Life Quality Studies, University of Bologna, Campus of Rimini, 47921 Rimini, Italy

**Keywords:** Physical inactivity, Children, COVID-19, Accelerometer, Sedentary behaviour

## Abstract

**Background:**

The World Health Organization stated an average of 60 min of Moderate to Vigorous Physical Activity (MVPA) that children should accumulate every day.

Nevertheless physical inactivity is growing and, due to restrictions imposed during pandemic, PA levels of children might be more negatively affected.

The study aimed to analyse the impact of COVID-19 on the PA of an Italian sample of primary school children by comparing it before and during COVID-19 considering gender differences.

**Methods:**

A pre-post analysis (October 2019–January 2021) was conducted using a randomized sample (*N* = 77) from the I-MOVE study settled in an Italian primary school. Both objective (Actigraph accelerometers) and self-reported (PAQ-c questionnaires) assessments of PA were performed. Changes were compared using T-Student and Chi-Square test. Gender differences were calculated using Anova.

**Results:**

Weekly and daily minutes time spent in MVPA significantly decreased respectively by − 30.59 ± 120.87 and − 15.32 ± 16.21 from before to during pandemic while the weekly time spent in sedentary behaviour increased (+ 1196.01 ± 381.49). PAQ-c scores followed the same negative trend (− 0.87 ± 0.72). Boys seem to have suffered more than girls from the imposed restrictions.

**Conclusion:**

These findings outline the need for strategies to promote PA and reduce sedentary behaviours in children to prevent COVID-19 restriction long-term effects.

## Background

The benefits of physical activity (PA) for children’s health and well-being are well-known [1, 2]. A growing body of evidence shows that greater amount and higher intensity of PA during childhood is associated with multiple beneficial outcomes such as cardiorespiratory and muscular fitness, cardiometabolic and bone health, academic performance, cognitive function, and mental health [3].

As many of these benefits are observed with an average of 60 min of moderate-to-vigorous daily PA, the updated “World Health Organization guidelines on physical activity and sedentary behaviour”, published in November 2020, confirmed that this is the minimum dose of PA that children should accumulate every day of the week [4].

Despite this evidence, prevalence estimates from intercontinental PA surveillance data are consistent in finding an insufficient level of PA in children across the world particularly evident among girls in comparison with boys of the same age [5, 6].

In European countries Steene-Johannessen J. at al., using accelerometers as PA objective measures, found that only up to 29% of children are categorized as sufficiently physically active, performing an average of at least 60 min PA per day, with substantial region-specific differences. In particular, the prevalence of adequately active children was higher in Northern (31%), intermediate in Central (26%), and significantly lower in Southern Europe (23%) [6]. Considering gender differences, objectively measured through Actigraph, boys were more active (13 min MVPA/day) and spent less time in sedentary behaviour compared to girls (8 min/day) [6].

The non-achievement of PA guidelines, referred to by the term of physical inactivity (PI) [7], is an established risk factor that has been identified as an important leading cause of death worldwide and already defined, in 2012, as a pandemic issue [8]. In connection, Katzmarzyk et al. has recently provided the most complete description of the global health burden associated with PI, finding that it is responsible for a total of 7.2% of all-cause deaths and for a substantial proportion of non-communicable diseases, ranging from 1.6% for hypertension to 8.1% for dementia [9].

Emerging data indicate that, as a consequence of the policies aimed at controlling the spread of the coronavirus disease 2019 (COVID-19), there has been a substantial increase in global PI levels in all age groups [10–13].

Thus, it has been hypothesized that the COVID-19 pandemic can exacerbate the pandemic of PI [14, 15].

Concerning the impact of COVID-19 on children’s PA habits, Yomoda et al. have conducted a scoping review finding a significant decline in PA among children [12]. In particular, the review’s results showed that the decrease was more prevalent among boys and older children and in those who live in apartments or houses with limited spaces and urban areas. This may be related to the fact that boys are used to practice organized team sports, most of which were stopped during the pandemic [16].

The 21 studies included in this review presented data related to the first half of 2020, when schools closure and lockdown measures were adopted almost worldwide. One of the highest school closure rates was reached at the end of March 2020 when schools were closed in 167 countries, affecting 82.8% of the world’s learners (more than 1.4 billion children and adolescents) https://en.unesco.org/covid19/educationresponse#schoolclosures.

As schools provide children with several opportunities for being physically active, such as physical education class and school playtime, [17, 18] a reduction in PA during the first wave of COVID-19 could be expected. Healthy behaviours such as active commuting to schools (i.e., walking or cycling), which represent a strategy to raise physical activity levels, have been restricted during COVID-19 and it was difficult to suppose that those healthy habits could be compensated at home [19]. Furthermore, during this period, other restrictions such as the closure of playgrounds, parks, recreational and sport facilities reduced the possibilities to be engaged in both structured and unstructured PA [20]. Finally, adolescents with lower physical fitness were observed to have further reduced physical activity levels during the pandemic [19].

Regarding cardio fitness status, Lopez et al. have conducted an important study focused on the effect of COVID-19 confinement on VO2 max findinga general small decrease in VO2 max with a statistically significant reduction only for adolescent girls [21]. Although short-term changes in children’s PA levels due to COVID-19 were found, to our knowledge, little is known about changes over a longer period and after the first peak of COVID-19 pandemic. Thus, in the most pessimistic scenario, it could be postulated that PA levels will continue to decline, determining even worse conditions than before the pandemic [12]. In this framework, at the current stage of the pandemic, it is fundamental to investigate the long-term effects of the COVID-19 on children’s PA [12]. Moreover, another important gap in the literature is that most of the studies examining physical activity in children during the COVID-19 pandemic didn’t use objective measurements [12]; for this reason, we believe that our study presents an added value.

Therefore, the present study aims to analyse the impact of COVID-19 on the PA of an Italian sample of primary school children by comparing PA levels before and after the re-opening of schools during COVID-19 pandemic also considering gender differences.

## Materials and methods

### Study design and participants

The present study was a pre-post analysis, using a randomized sample from the I-MOVE study settled in a primary school of a northern Italian city (Imola, Emilia Romagna Region).

The I-MOVE study was endorsed by the University of Bologna (Italy). Approval for the study was obtained from the University of Bologna Bioethics Committee, on 18 March 2019 (Prot. n. 0054382 of 18 March 2019 (UOR: SI017107-Classif. III/13)). The study was conducted following the Declaration of Helsinki. Informed consent was obtained from all parents and/or legal guardian(s) of the participants.

### Data collection and outcomes

Baseline assessment was completed in October 2019 and a second intermediate assessment was performed in January 2021 after 1 year of COVID-19 pandemic.

Socio-demographic information was obtained during the baseline assessment. Parents’ education level was stratified into three categories: low (completed primary and middle school), medium (high school diploma) and high (university degree).

In October 2019, anthropometric characteristics were collected following standard procedures [22, 23] by the study research staff. In particular, height was measured to the nearest 0.1 cm using a portable stadiometer (SECA 217; Hamburg, Germany), body weight was measured to the nearest 0.1 kg (light indoor clothing, without shoes) using a calibrated electronic scale (SECA 877; Hamburg, Germany).

Considering the strict school rules during the pandemic and the importance of maintaining physical distancing, the research team could not carry out anthropometric measurements during the second intermediate assessment. Hence, the parents of children participating in the I-MOVE study remotely self-report anthropometric characteristics of their child, i.e. height and weight, using an online questionnaire in January 2021.

Body-Mass Index (BMI) was calculated as weight (in kilograms) divided by the square of height (in meters). This index was used to assess children’s weight status according to Cole cut off values by sex and age [24, 25].

PA outcomes were monitored through self-reported and objective measures both in October 2019 before the COVID-19 and in January 2021 during the COVID-19 pandemic when schools were re-opened. Self-reported physical activity questionnaires for children (PAQ-C) were used to investigate PA during school time, leisure time and PA during organized sports. The PAQ-C is a self-administered, 7-day recall questionnaire, with 9 items scored on a five-point scale. This instrument yielded a final composite activity score by calculating the mean of the 9 items. This questionnaire has been shown to be valid and reliable [26].

Objective PA and time spent in sedentary behaviours (SB) data were assessed using an accelerometer actigraph (Actigraph, LLC, Pensacola, FL, USA) (ActiLife6 wGT3X-BT set to 10-s epochs). The actigraph assessment was carried out following a careful sanitization of the instrument before and after use.

The children were instructed to wear the actigraph on their right hip using a specific waistband [27] over a seven-day period (five weekdays and two weekend days), with the exclusion of water activities (e.g., showering, swimming).

Actigraph data were examined through ActiLife 6.13.3 software (ActiGraph, LCC, Pensacola, FL, USA), with an epoch length of 10 s to allow a more detailed estimate of PA intensity [28].

Valid wear time was defined based on a specific inclusion criterion: having worn the accelerometer for at least 10 h every day (sleeping hours included) during at least 3 weekdays and 1 weekend day.

The cut points by Evenson were used to calculate the minutes spent per type of physical activity (light, moderate and vigorous) per day [29].

### Statistical analysis

Data analysis was carried out using SPSS, version 22 (Statistical Package for Social Science) (SPSS Inc. Chicago, IL, USA). Continuous variables are presented as means and standard deviation (SD), and categorical variables are presented as frequency (percentage).

Considering the normal distribution of our sample, verified by the Esplora SPSS function, we analysed differences in PA outcomes, both objective and self-reported, before and during COVID-19 within groups, using the paired-samples t-test for continuous variables and the Chi-square test for categorical ones.

Gender subgroup analysis was performed using one-way ANOVA. Significance level was set to *p* < 0.05.

## Results

### Sample description

A total of *N* = 77 children was randomized within the I-MOVE study and enrolled in the present analysis. Table 1 shows the general demographic characteristics of the sample, collected in October 2019, before the COVID-19 pandemic. Children’s ages ranged from 7 to 10 years, with an average age of 7.84 (SD 1.41) and BMI of 17.80 (SD 2.82). The majority of the sample was male (60.80%). The most prevalent parents’ education level reported was medium.

**Table 1:** Sample characteristics

Variables	N (*N* = 77)	Mean ± SD or %
Age (n, years)	77	7.83 ± 1.42
Male (n, %)	48	62.3%
Female (n, %)	29	37.7%
BMI Total (n, score)	78	17.81 ± 2.85
Mother Education		
Low (n, %)	12	18.2%
Medium (n, %)	31	47.0%
High (n, %)	23	34.8%
Father Education		
Low (n, %)	17	25.8%
Medium (n, %)	36	54.5%
High (n, %)	13	19.7%

*BMI* body max index

### Physical activity levels

Table 2 describes changes between before and during COVID-19.

**Table 2:** Changes between before and during the COVID-19 pandemic

Variables	Before COVID-19 Mean ± SD or %	During COVID-19 Mean ± SD or %	Changes Mean ± SD	***P*** Value^*^
Age (years)	7.84 ± 1.41	9.19 ± 1.41	+ 1.35 ± 0.03	< 0.0001*
BMI (score)	17.49 ± 2.76	17.91 ± 3.00	+ 0.10 ± 0.21	0.05*
BMI Cole cut-off				< 0.001*
Normal weight (n, %)	54 (71.1%)	52 (68.4%)		
Overweight (n, %)	16 (21.1%)	20 (26.3%)		
Obese (n, %)	6 (7.9%)	4 (5.3%)		
Weekly MVPA (min)	332.94 ± 118.42	301.95 ± 109.81	−30.59 ± 120.87	0.01*
Daily MVPA (min)	55.44 ± 19.1	40.13 ± 14.18	−15.32 ± 16.21	< 0.001*
Adhering to MVPA guideline of 60 min/d; (n, %)	33 (42.9%)	9 (11.7%)		< 0.001*
Not adhering guideline	44 (57.1%)	68 (88.3%)	*N* = −24	< 0.001*
Sedentary Activity (min/week)	6605.88 ± 417.30	7801.89 ± 409.92	+ 1196.01 ± 381.49	< 0.001*
Step Counts (*n*/week)	54,687.39 ± 13,015.37	51,534.86 ± 11,615.04	− 3152.53 ± 11,433.77	0.02*
Light (min/week)	1711.06 ± 308.33	1694.92 ± 297.54	−16.16 ± 267.67	0.60
Moderate (min/week)	219.79 ± 72.26	203.99 ± 68.00	−15.80 ± 65.86	0.04*
Vigorous (min/week)	113.15 ± 53.40	97.95 ± 47.36	− 15.19 ± 46.06	0.005*
Physical Activity Levels (PAQ-C score) *N* = 52	3.06 ± 0.75	2.19 ± 0.57	−0.87 ± 0.72	< 0.001*

Changes in continuous measures between before and during pandemic were compared using T Student for paired sample test and Chi- Square test for dichotomous variables

*MVPA* moderate to vigorous physical activity, *BMI* body-mass index, *PAQ-c* physical activity questionnaire for children

*Significant *p*-value < 0.05

From 2019 to 2021 sample’s age and BMI, as expected, increased significantly.

BMI score increased on average within the normal range, while the children’s distribution in the different weight categories, based on the International Obesity Task Force (IOTF) cut-offs, varied with a significant reduction of children with normal-weight (71.1% versus 68.4%) and increase of children with over-weight (21.1% versus 26.3%).

All actigraph parameters significantly worsened: weekly and daily minutes spent in PA significantly decreased by 30.59 and 15.32 min, respectively, from before to during COVID-19 (*p* value ≤0.01).

A similar decrease was observed in the duration of the time spent in PA of different intensity: light (− 16.16 ± 267.67), moderate (− 15.80 ± 65.86) and vigorous (− 15.19 ± 46.06), with a significant decrease for both moderate and vigorous PA whereas light PA did not decrease significantly. The same reduction was also observed for the weekly step count. Overall, in October 2019, 57.1% of the sample did not reach the recommended level of PA, and this percentage increased significantly in January 2021, with a total of 88.3% of children not meeting the recommended levels of PA.

By contrast, a significant increase of minutes (+ 1196.01 ± 381.49) spent in sedentary activities was observed.

The self-reported PA measurement, assessed with the PAQ-c questionnaire and completed by *N* = 52 children, followed the same trend of the objective actigraph measurement. The PAQ-c total score significantly decreased from October 2019 to January 2021 (− 0.87 ± 0.72).

### Gender differences

Gender differences were observed for both objective and self-reported measures of PA during the COVID-19 pandemic as compared to the same period 1 year and a half before (Table 3).

**Table 3:** Gender differences between before and during COVID-19 pandemic (*N* = 77)

Variables	Before COVID-19		During COVID-19		Before and During COVID-19 Changes Mean ± SD		***P*** value*
	Boys (***N*** = 48)	Girls (***N*** = 29)	Boys (***N*** = 48)	Girls (***N*** = 29)	Boys (***N*** = 48)	Girls (***N*** = 29)	
Weekly MVPA (min)	368.69 ± 116.58	273.76 ± 97.11	316.60 ± 114.87	277.70 ± 98.00	−52.09 ± 110.46	+ 3.94 ± 85.89	0.02*
Daily MVPA (min)	61.38 ± 19.58	45.63 ± 16.18	41.83 ± 14.70	37.30 ± 13.01	−19.54 ± 16.55	−8.32 ± 13.13	0.003*
Not meeting the recommended levels (<60minper day MVPA)	21 (43.75%)	23 (79.31%)	41 (85.42%)	27 (93.10%)	*N* = + 20	*N* = + 4	0.02* (girls) ns (boys)
Weekly sedentary activities (min)	6532,40 ± 392.26	6727.52 ± 435.67	7794.82 ± 359.03	7813.60 ± 489.38	+ 1262.42 ± 386.60	+ 1086.08 ± 350.39	0.05*
PAQ-c total score	3.34 ± 0.72	2.69 ± 0.78	2.25 ± 0.60	2.08 ± 0.64	−1.10 ± 0.80	−0.60 ± 0.52	0.01*

*MVPA* moderate to vigorous physical activity, *PAQ-c* physical activity questionnaire for children

^*^Changes between before and during pandemic were compared using OneWay-Anova stratifying results by gender for continuous measures and Chi-square test for dichotomous variables

Weekly time spent in PA significantly decreased significantly in boys (−52.09 ± 110.46) while girls did not undergo a substantial decrease over the total PA but, on the contrary, there was a slight increase in the weekly PA from before to during the COVID-19 pandemic. Both groups reduced the daily PA, especially boys (− 19.54 ± 16.55), and the number of children who did not reach the PA guidelines of ≥60 min/d of PA increased in our sample with significant values for girls. Before the pandemic, girls in our sample had very low daily levels of MVPA (45.63 ± 16.18) and the 79.31% did not meet the recommended levels of PA. The COVID-19 pandemic has further exacerbated this condition with 93.10% of girls in January 2021 who reported levels of MVPA lower than 60 min per day. Considering the sedentary behaviour before COVID-19, girls were more sedentary than boys (girls: 6727.52 ± 435.67 vs boys: 6532,40 ± 392.26) and during the pandemic the increasing sedentary lifestyle reached higher values in the girls (girls: 7813.60 ± 489.38 vs boys: 7794.82 ± 359.03).

Generally, both boys and girls rose their time spent in sedentary activity of + 1262.42 ± 386.60 and + 1086.08 ± 350 min per week, respectively, with a greater increase for boys. Finally, also the PAQ-c total score calculated from before to during COVID-19 was significantly lower in boys than girls.

## Discussion

The present study evaluated the impact of COVID-19 restrictions on PA levels in an Italian sample of primary school children comparing data from October 2019 to January 2021.

After China, Italy was the second country in the world that experienced the impact of COVID-19 with a high COVID-19 associated mortality, particularly in the early months of the pandemic [30]. For this reason, Italy was the first European country to enact measures for school closure and to implement a national lockdown to contain the spread of COVID-19 [31]. In particular, in Italy, schools were closed on 5th March 2020 and re-opened on 14th September 2020; during this period, education was not interrupted but continued online. After this period, primary schools remained open, except between 8th March 2021 and 7th April 2021 (1 month of distance learning).

In our study, all children obtained a general reduction in all actigraph parameters. A larger impact was found among boys, who reduced their PA levels significantly more than girls, who showed a smaller decrease. However, it should be taken into account that girls had generally lower levels of MVPA before COVID-19 and a lower percentage met the WHO recommendations about the amount of PA every day; in January 2021 there was a further deterioration of this inequalities..

Our results are in line with recent literature regarding the impact of the pandemic restriction on children’s PA levels [12, 13, 32, 33]..

In particular, Yomoda et al., in their scoping review, summarized data related to the first half of 2020, when lockdown measures were in place in many countries, finding that COVID-19 pandemic caused a decline in PA among children, especially in boys and in older children/adolescent [12]. A cohort study conducted in Dutch primary school children found that, even after the lockdown measures and the re-opening of the schools (June 2020), PA still decreased, while screen and sedentary time increased [34].

Considering the Italian scenario, Pietrobelli et al. a study on children and adolescents with obesity, compared data from May–July 2019 to March–April 2020, and found 2.30 h/week lower exercise’s time [32]. This reduction is in line with another Italian study that evaluated, 15 days after the first lockdown (10th March 2020), the effects of the COVID-19 quarantine in youth. As expected, staying at home without the possibility to go outside changed many routines, and during the quarantine, only 15.5% of youth practiced at least 60 min of PA [35]. Our results confirm this negative trend and seem to suggest that COVID-19 could negatively affect the children’s PA levels even after 1 year since the beginning of the pandemic.

Another great alarm factor, in line with other studies [20, 32, 34], was the increasing amount of time spent in sedentary activities. As found by ten-Velde et al. in their study in Dutch primary school children, this increase, despite the re-opening of schools, does not point to a reduction.

The reduction in PA and the increase in SB is particularly alarming because, as previous research suggests, even brief periods of sustained physical inactivity can have detrimental effects on muscle mass, glucose homeostasis, cardiovascular function and structure, and increase cardiovascular risk factors [10, 36].

To our knowledge, this is the first Italian study to provide objective and self-reported PA data aiming at assessing the long-term effects of COVID-19 on children’s PA behaviours as recommended by Yomoda et al. [12] Moreover, in line with our findings, a recent systematic review with metanalysis performed by Runacres et al. found a strong impact of the COVID-19 pandemic on children’ sedentary time. However, all the included studies used only self-reported measurements to assess sedentary time and,furthermore, they were not focused on a longer impact of COVID-19 but on the effects of lockdown or similar restrictions (such as school closures or homestay requirements) [37]. Considering that PA habits in childhood are of great importance as children who are active in their youth are more likely to continue of being active into adulthood [38] and that the effects of COVID-19 could be prolonged, public health stakeholders should consider these findings to prevent the negative effects of PI among children by promoting strategies to restore PA to a sufficient level.

Parents, schools, health policymakers, and governments need to be aware of this situation and consider our findings in order to promote proactive strategies and interventions to improve PA levels and prevent the negative effects of PI [12, 33]. As structured settings, such as schools, afterschool programs, summer and sport camps have shown to provide substantial amounts of PA during attendance and to make an important contribution to the accumulation of youth health enhancing PA [18], these types of settings could be favourable environments for pursuing this aim. Furthermore, school time and physical education lessons should make the difference and provide both adequate environments and support to encourage children to be physically active [17].

Thus, classroom PA programs should be included in school health guidelines as an integrative approach in cooperation with educative stakeholders to re-establish the recommended level of PA and reduce the increase in sedentary behaviour [39].

Our study presents some limitations. Firstly, the use of a small sample from an ongoing study that could represent a bias. Although this population might not be representative of the Italian primary school population but only of a region with a good culture on active lifestyles, our findings provide a reliable estimation of the PA patterns of primary school children. Moreover, few studies used both objective and self-reported measures to assess PA levels [34]. Although the PAQ-c questionnaire has been proved to be an acceptable and reliable instrument [26], some concerns remain about the variability of the self-assessments conducted by the participants [40]. Furthermore, previous research [34] showed that seasonal variations could affect this type of results; nevertheless, we performed both the assessments in autumn and winter in order to make pre- and post-assessments comparable in terms of seasonal period. We did not perform the analysis considering age and BMI sub-categories, this could represent a study limitation. However, during baseline assessment, no statistically significant differences were found by stratifying by BMI and age; furthermore, given the lower sample size, we preferred to keep the sample united rather than proceeding with many sub-analyses.

In addition, we found a slight decrease in children with normal weight, but because during the COVID-19 pandemic, anthropometric measures were reported by parents and not collected by research staff, the interpretation of this result should be cautious.

## Conclusion

The present study provides an insight into the potential impact of COVID-19 on children PA behaviour in a sample of Italian primary school children. During COVID-19 pandemic, even after the re-opening of schools, all children reduced their PA levels and increased sedentary habits, as reported by both objective and self-reported measures. The decrease was especially high in boys suggesting that they have been disproportionally affected by lockdown restrictions suggesting that probably they have been disproportionally influenced by lockdown restrictions.

These findings highlight the need for strategies to promote PA and reduce sedentary behaviours in children to prevent COVID-19 restriction long-term effects. These reflections confirm the need to create educational networks (family, school, sport and recreational environment) connected to each other to address the problem of child sedentarism.

## Data Availability

The datasets used and/or analysed during the current study are available from the corresponding author on reasonable request.

## Abbreviation

BMI: Body Mass Index
COVID-19: Coronavirus disease 2019
IOTF: Obesity Task Force
MVPA: Moderate Vigorouse Physical Activity
PA: Physical Activity
PAQ-c: Physical Activity Questionnaires for Children
PI: Physical Inactivity
SB: Sedentary Behaviour
